# Supplementary material for: The impact of feature selection on one and two-class classification performance for plant microRNAs
Source: PeerJ. 2016 Jun 21;4:e2135. doi: 10.7717/peerj.2135 (PMC4924126; doi:10.7717/peerj.2135)
Supplement: File S1 [file peerj-04-2135-s001.pptx]

## Slide 1
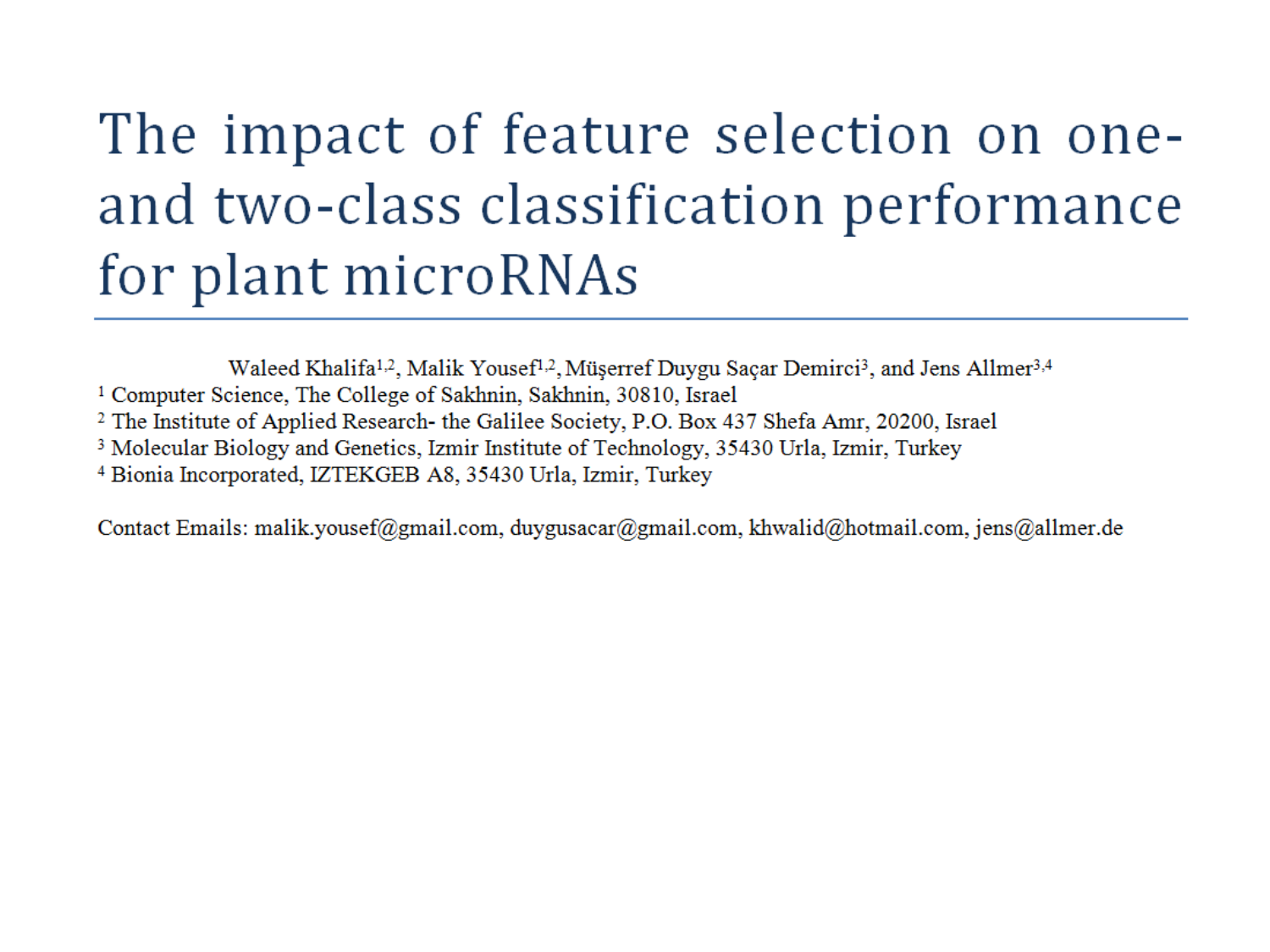

## Slide 2
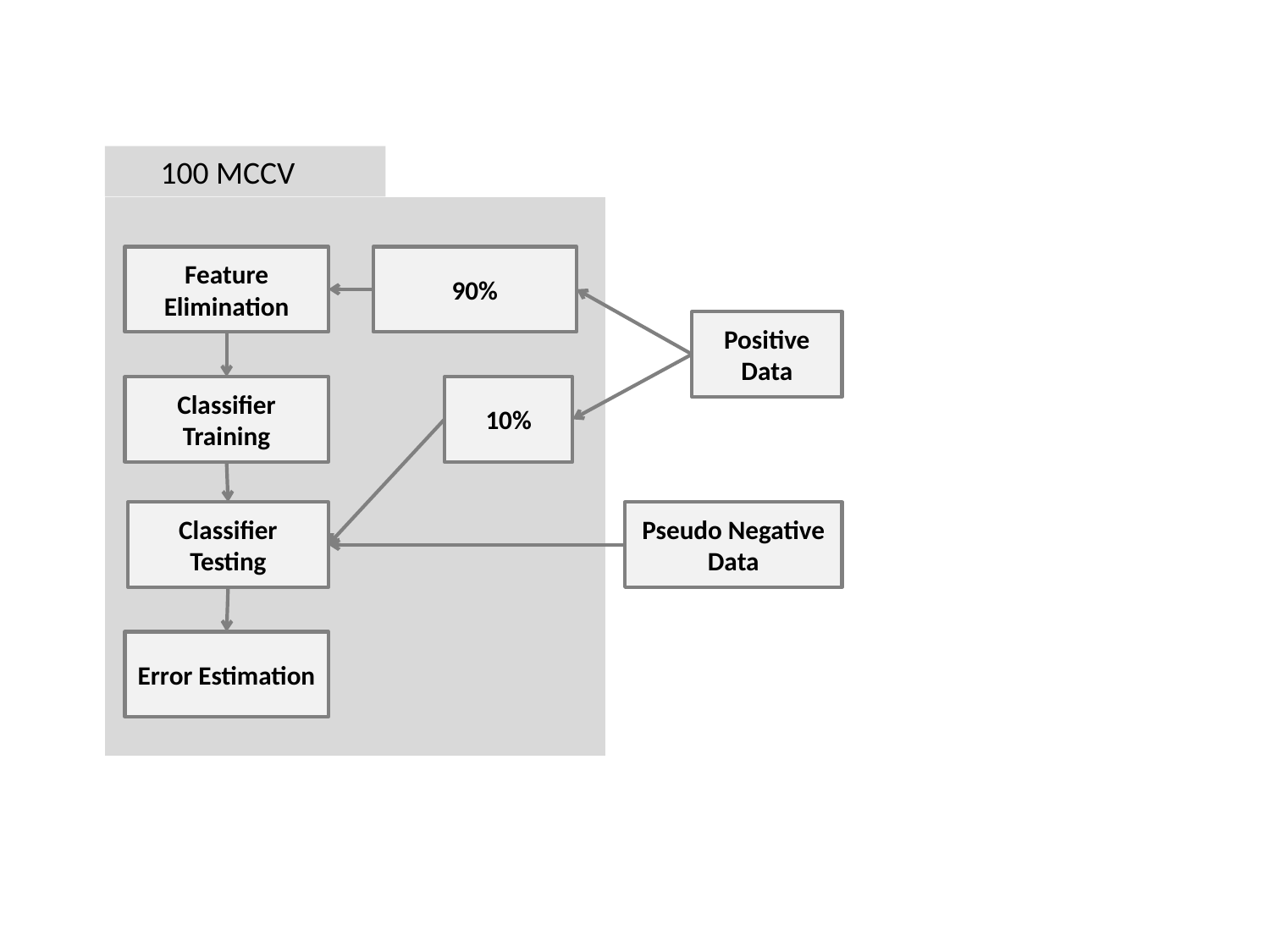

100 MCCV
Feature Elimination
90%
Positive Data
Classifier Training
10%
Classifier Testing
Pseudo Negative Data
Error Estimation

## Slide 3
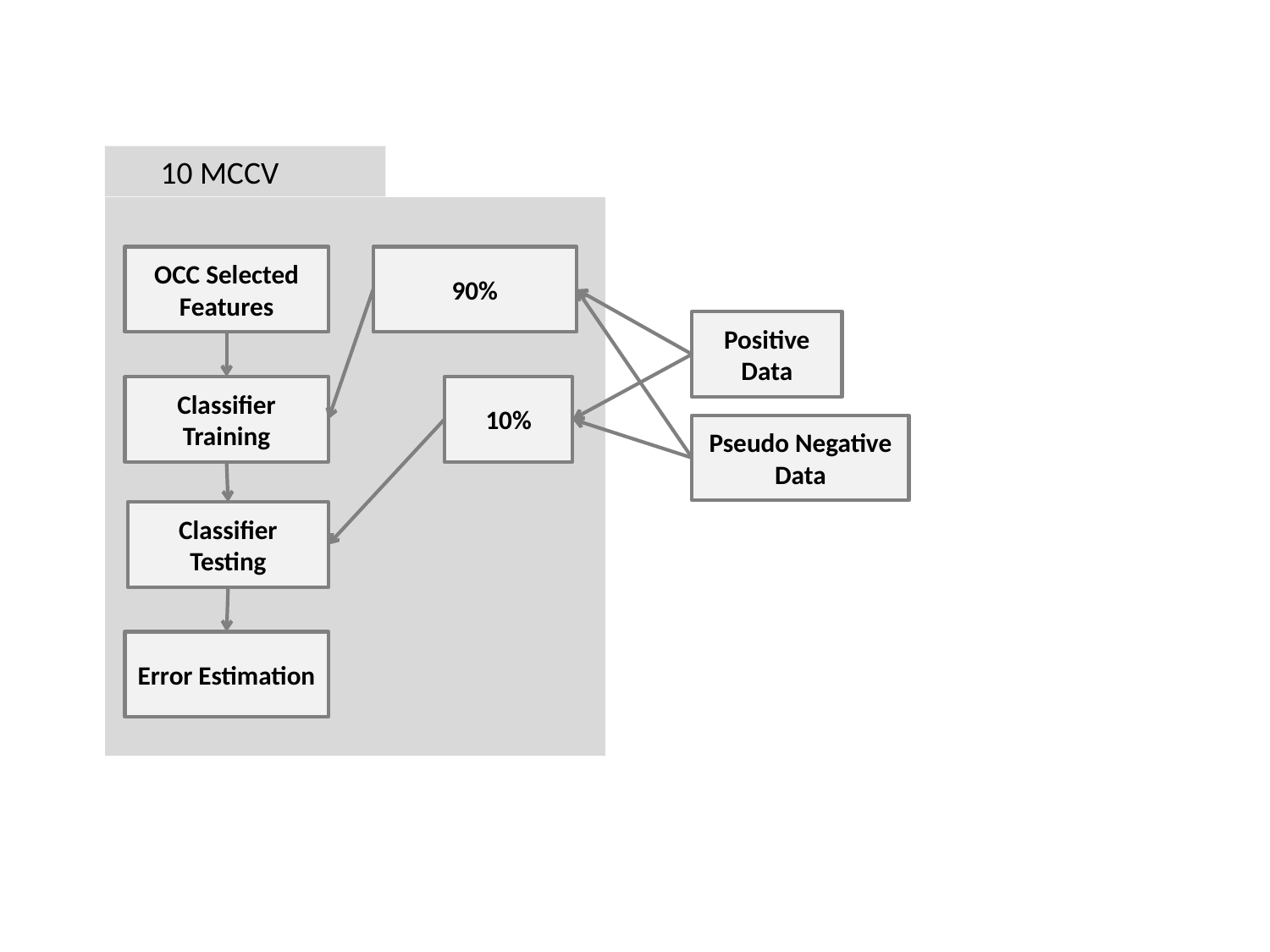

10 MCCV
OCC Selected Features
90%
Positive Data
Classifier Training
10%
Pseudo Negative Data
Classifier Testing
Error Estimation

## Slide 4
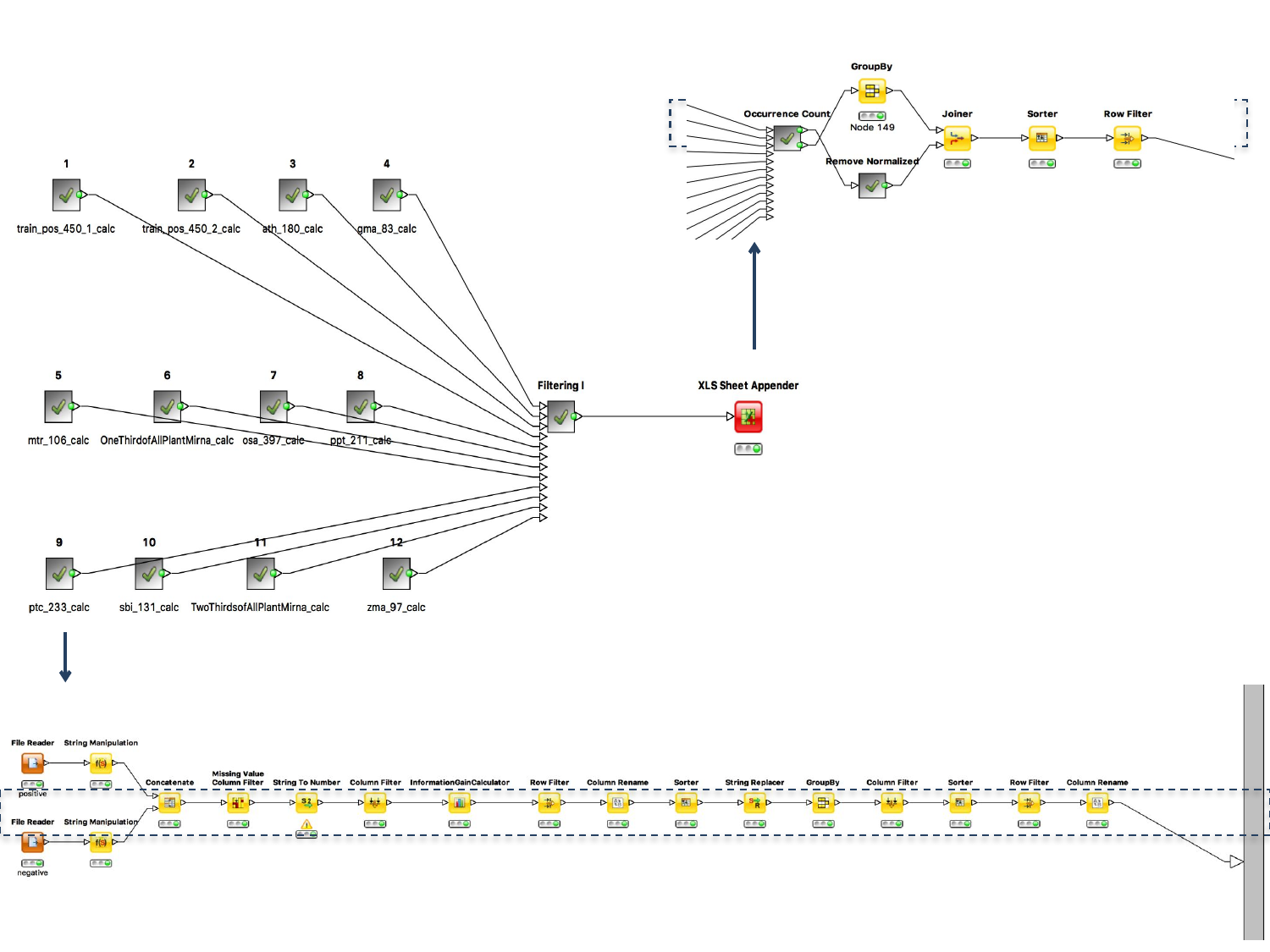

## Slide 5
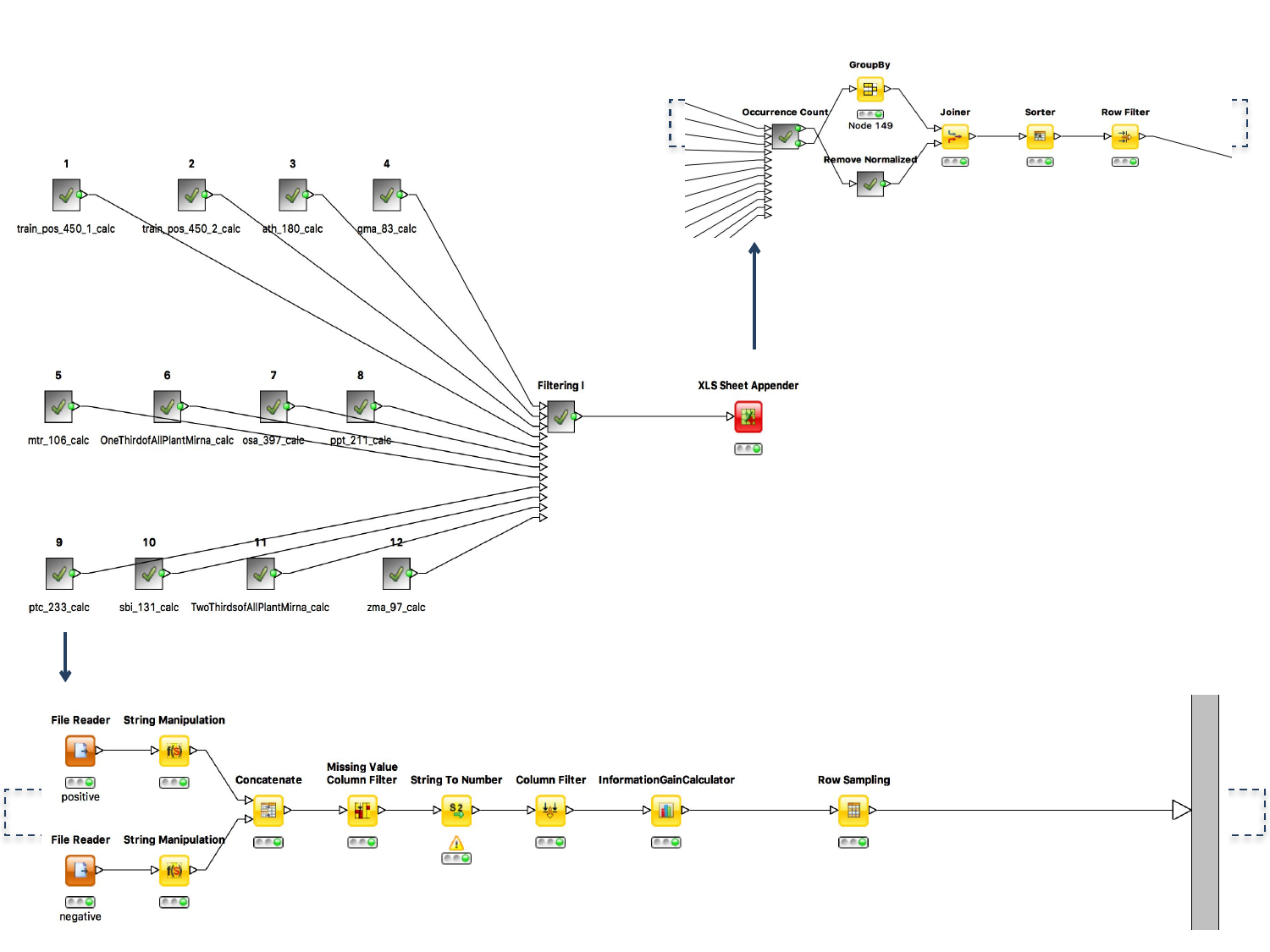

## Slide 6
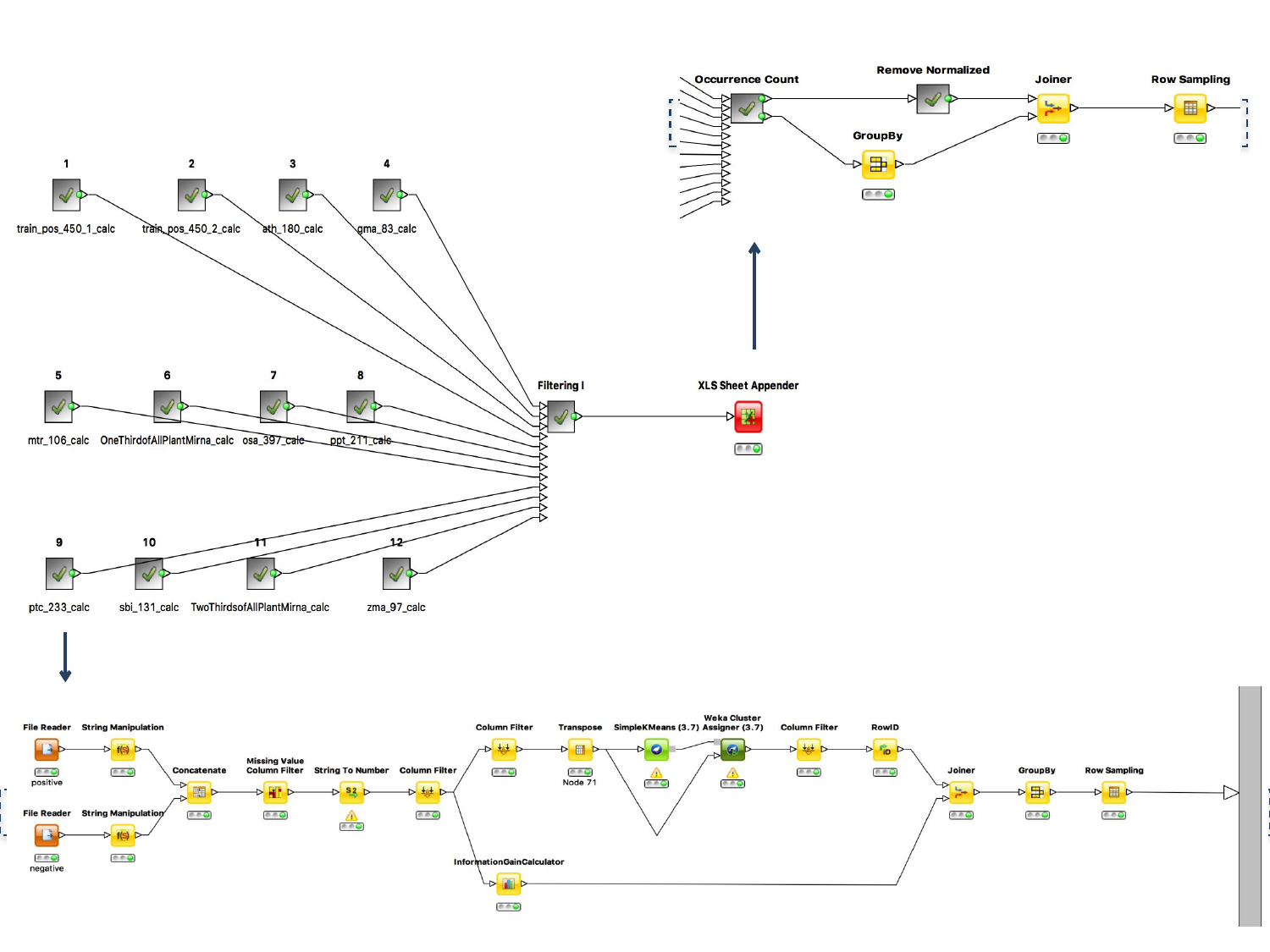

## Slide 7
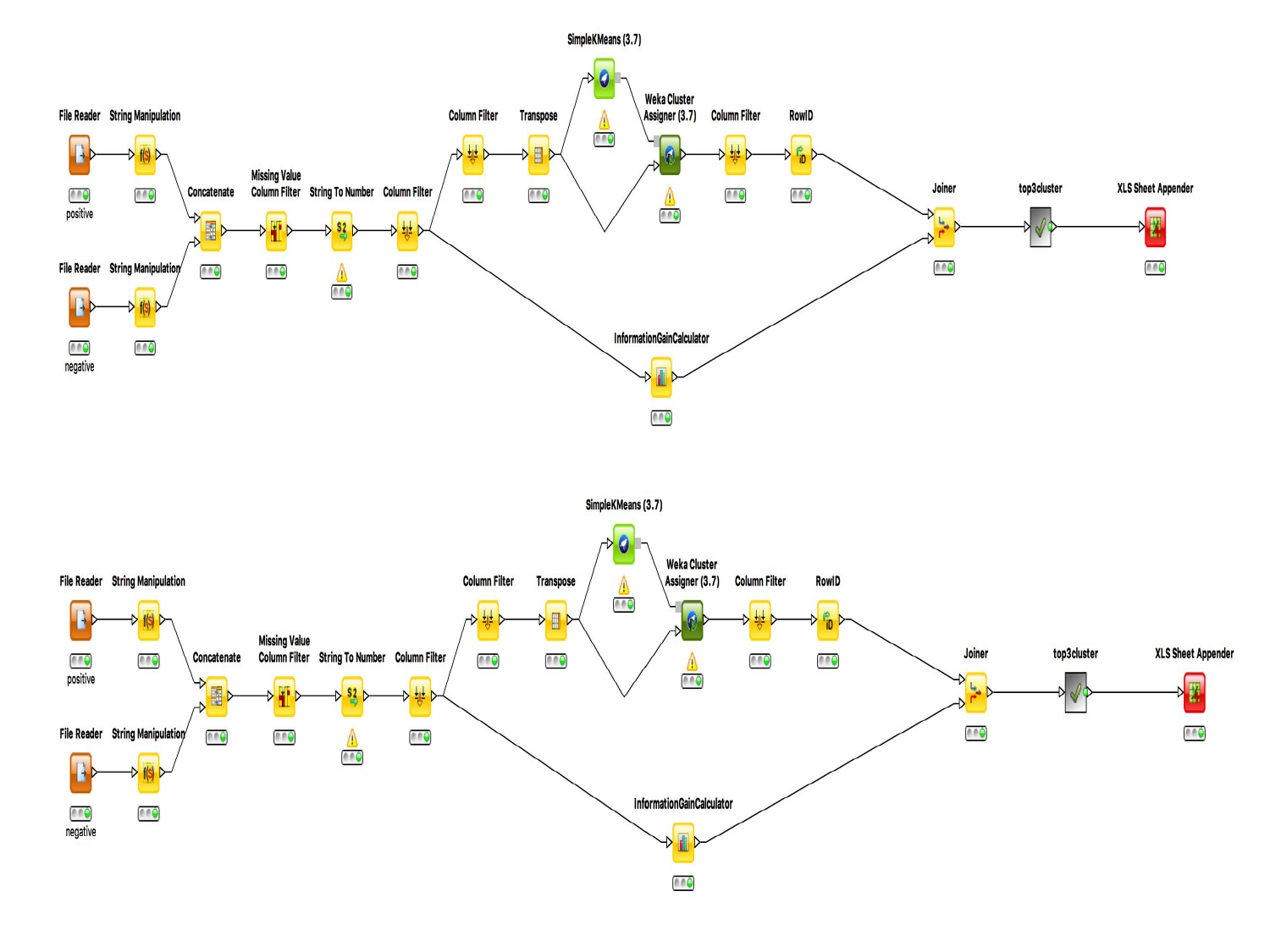

## Slide 8
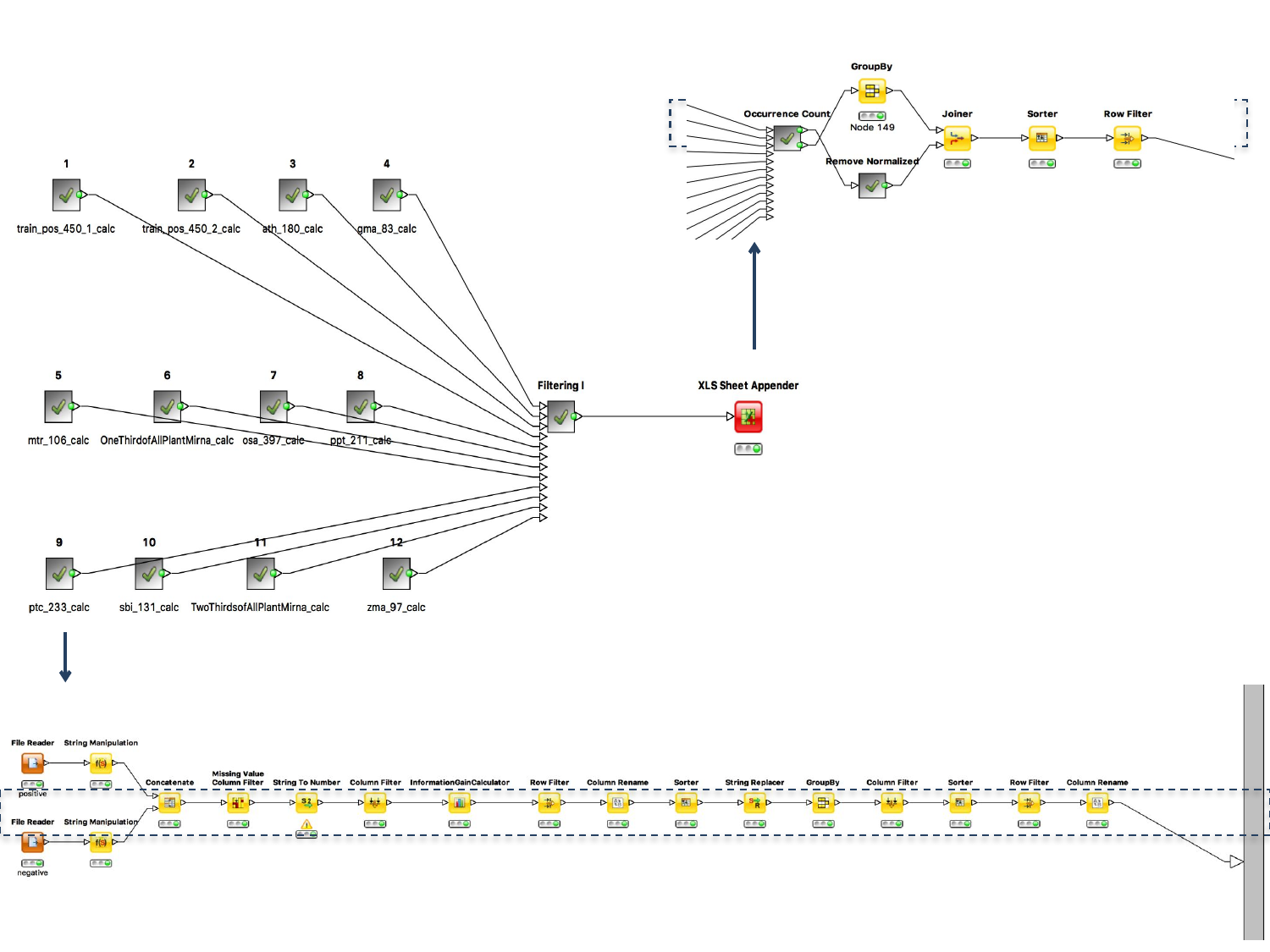

## Slide 9
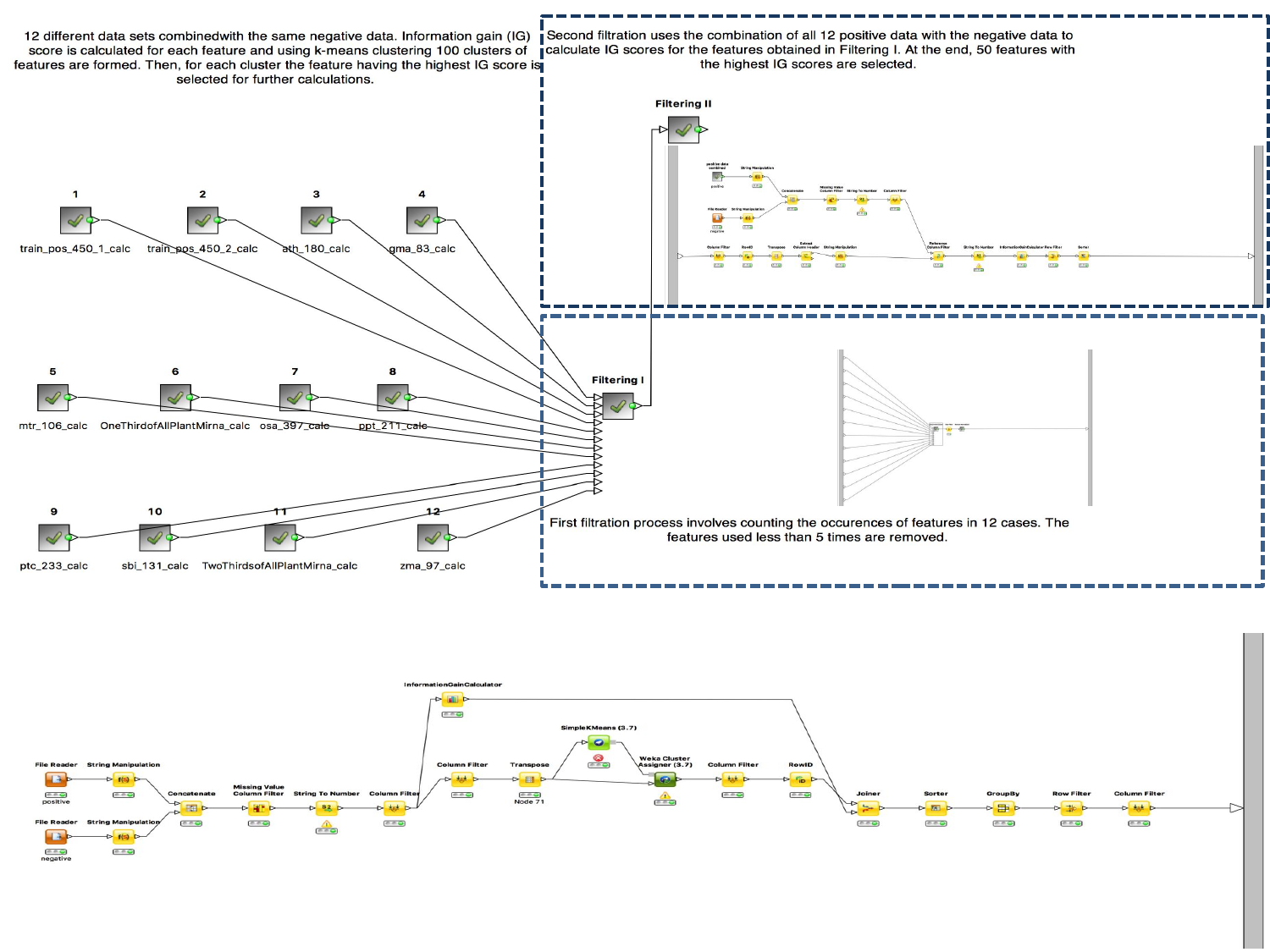

## Slide 10
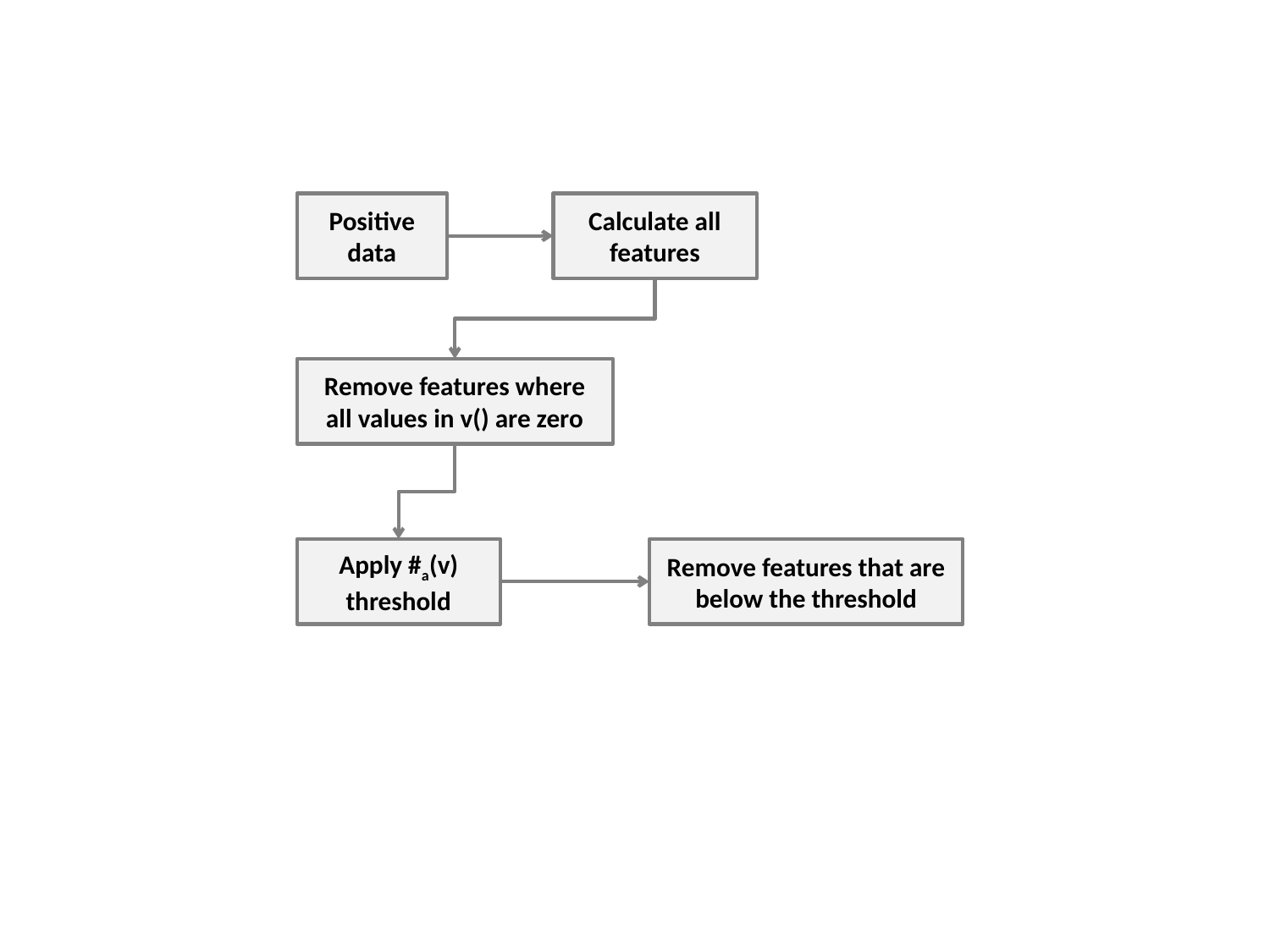

Positive data
Calculate all features
Remove features where all values in v() are zero
Apply #a(v) threshold
Remove features that are below the threshold

## Slide 11
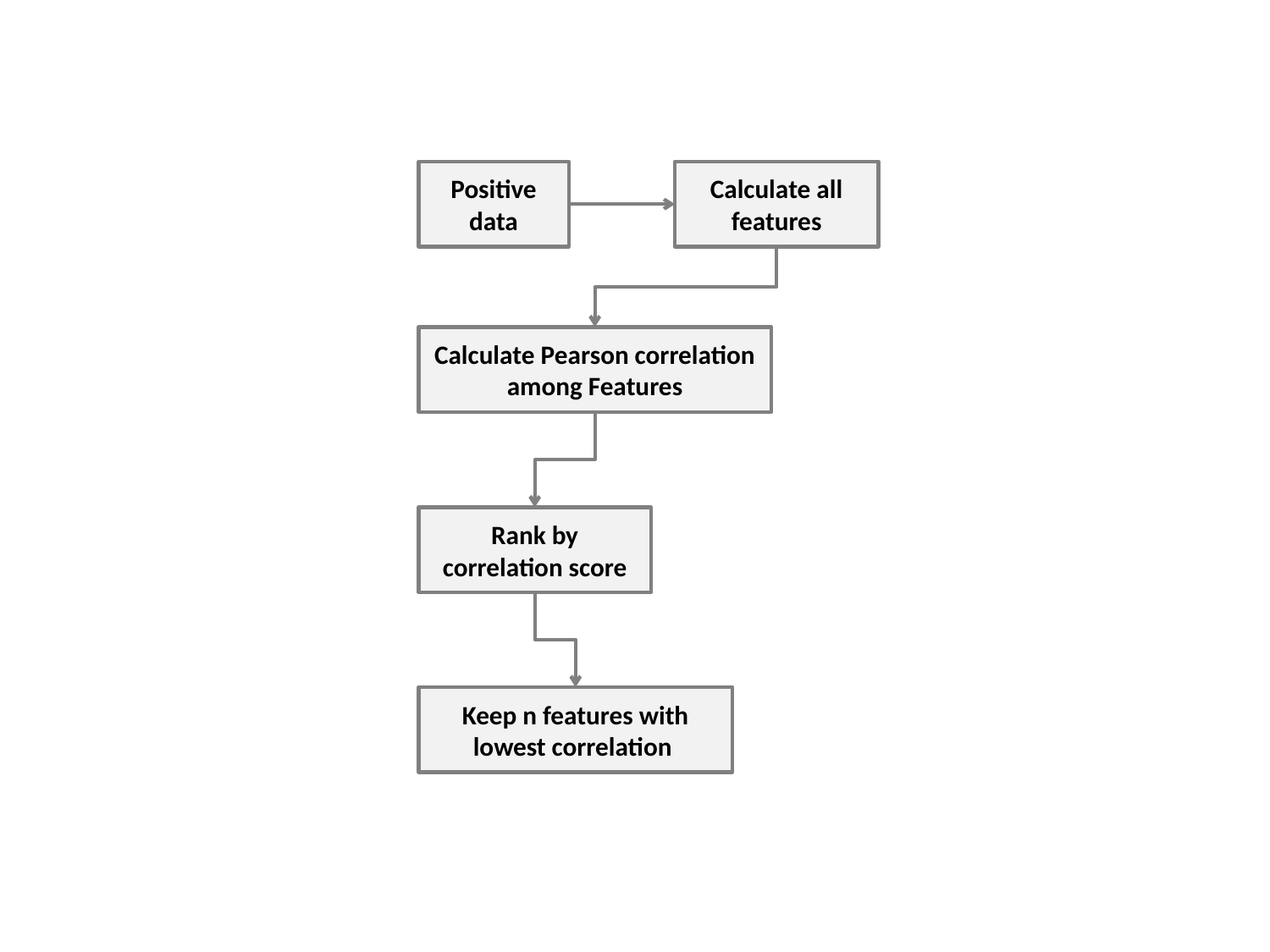

Positive data
Calculate all features
Calculate Pearson correlation among Features
Rank by correlation score
Keep n features with lowest correlation
